# Simple Technique for in Field Samples Collection in the Cases of Skin Rash Illness and Subsequent PCR Detection of Orthopoxviruses and Varicella Zoster Virus

**DOI:** 10.1371/journal.pone.0096930

**Published:** 2014-05-19

**Authors:** Catherine Dumont, Leonid M. Irenge, Edmond K. Magazani, Daniel Garin, Jean-Jacques T. Muyembe, Mostafa Bentahir, Jean-Luc Gala

**Affiliations:** 1 Royal Military Academy, Bruxelles, Belgium; 2 Defense Laboratories Department, ACOS Ops&Trg, Belgian Armed Forces, Peutie, Belgium; 3 Center for Applied Molecular Technologies, Institut de Recherche Expérimentale et Clinique, Université catholique de Louvain, Brussels, Belgium; 4 Ministère de la Santé Publique, Gombe, République Démocratique du Congo; 5 IRBA, Institut de Recherches Biomédicales des Armées, Service de Santé des Armées, Bretigny-sur-Orge, Cedex, France; 6 Laboratoire National de Santé Publique, Institut National de Recherche Biomedicale, Kinshasa, Democratic Republic of Congo; CEA, France

## Abstract

**Background:**

In case of outbreak of rash illness in remote areas, clinically discriminating monkeypox (MPX) from severe form of chickenpox and from smallpox remains a concern for first responders.

**Objective:**

The goal of the study was therefore to use MPX and chickenpox outbreaks in Democratic Republic of Congo (DRC) as a test case for establishing a rapid and specific diagnosis in affected remote areas.

**Methods:**

In 2008 and 2009, successive outbreaks of presumed MPX skin rash were reported in Bena Tshiadi, Yangala and Ndesha healthcare districts of the West Kasai province (DRC). Specimens consisting of liquid vesicle dried on filter papers or crusted scabs from healing patients were sampled by first responders. A field analytical facility was deployed nearby in order to carry out a real-time PCR (qPCR) assay using genus consensus primers, consensus orthopoxvirus (OPV) and smallpox-specific probes spanning over the 14 kD fusion protein encoding gene. A PCR-restriction fragment length polymorphism was used on-site as backup method to confirm the presence of monkeypox virus (MPXV) in samples. To complete the differential diagnosis of skin rash, chickenpox was tested in parallel using a commercial qPCR assay. In a post-deployment step, a MPXV-specific pyrosequencing was carried out on all biotinylated amplicons generated on-site in order to confirm the on-site results.

**Results:**

Whereas MPXV proved to be the agent causing the rash illness outbreak in the Bena Tshiadi, VZV was the causative agent of the disease in Yangala and Ndesha districts. In addition, each on-site result was later confirmed by MPXV-specific pyrosequencing analysis without any discrepancy.

**Conclusion:**

This experience of rapid on-site dual use DNA-based differential diagnosis of rash illnesses demonstrates the potential of combining tests specifically identifying bioterrorism agents and agents causing natural outbreaks. This opens the way to rapid on-site DNA-based identification of a broad spectrum of causative agents in remote areas.

## Introduction

Orthopoxviruses (OPV) are large double-stranded DNA viruses which replicate exclusively in the cytoplasm of host cells [1], [2]. This genus is composed of several virus species, many of whom are pathogens for mammalians and/or humans. In humans, symptoms of orthopoxvirus infections range from mild skin lesions to fatal systemic disease, as is the case for the major form of smallpox. This disease is characterized by a generalized rash associated with a high mortality rate in unvaccinated persons [3]–[7].

Monkeypox (MPX) is an endemic zoonosis mainly arising in forest areas in Democratic Republic of Congo and its adjoining countries [8]–[10]. MPX in humans clinically mimics smallpox symptoms [9], [11], [12] but is less contagious and has a lower mortality rate [9], [13]. Typical MPX is characterized by a febrile prodrome, the development of lymphoadenopathy followed by a generalized rash with well-circumscribed lesions at the same stage of development and a pattern of centrifugal distribution. In contrast, varicella is a febrile rash illness characterized by the absence of a significant febrile prodrome and by lesions at different stages, with bumps, blisters, and scabbed lesions existing at the same time [14]. Regarding operational perspectives, diagnosis can be challenging because MPX atypical forms cannot always be easily distinguished from severe varicella [14]–[16] or from smallpox [17]. In case of presumed diagnosis of MPX in DR Congo, samples have to be collected and sent to the Laboratoire National de Santé Publique [LNSP] (Institut National de Recherche Biomedicale [INRB], Kinshasa) for viral identification. However, DR Congo is facing difficult challenges in terms of transport and communication. Kasai provinces which have both been the epicentres of emerging diseases for the past decades are not easily accessible by road, train or plane, a transport limitation which isolates them for major cities elsewhere in the country. Accordingly, shipment of samples to INRB-Kinshasa remains difficult and requires the contribution of the United Nations and the NGOs. Quick outbreak management (identification of the causative agent, mitigation, and containment of such outbreaks) is often hampered by the slow process affecting the chain of sample collection, transportation and analysis, leading to foreign intervention rather than local management by Congolese responders. The development of rapid sampling method and simple identification methods which can be easily implemented in the field could substantially improve rapid identification of causative agents, pending definitive confirmation by the LNSP or by any other reference laboratory. This potential was illustrated during 2008–2009 outbreaks of skin rash illness in West Kasai province, which were presumably attributed to MPXV. Accordingly, we report here the development of DNA-based assays for rapid identification of agents causing skin rash disease outbreaks and their use in the field in April 2009. These assays demonstrated their potential for rapidly identifying causative agents in human specimens sampled on blotting papers.

## Materials and Methods

### Deployment of DNA-based Diagnostic Facility in the Vicinity of Suspected MPK Outbreaks, in Kananga, DR Congo

Following reports of a rash illness outbreak presumably due to MPX in the Ndesha healthcare district, a team consisting of a medical doctor and two biologists equipped with a rapidly deployable DNA-based diagnostic capacity was sent to Kananga, the provincial capital.during the second week of April 2009. Two days after departure from the Belgian military airport of Meslbroek, the tent was deployed inside the Kuya Kumpala Belgian military camp located in the outskirts of Kananga, the provincial capital. The laboratory consisted of a rapidly deployable DNA-based diagnostic capacity with the minimum equipment required for safe handling and processing of clinical specimen, i.e., samples reception and inactivation through DNA or RNA extraction, a single-use portable glove box, a real-time PCR (qPCR) and the UV transilluminator and electrophoresis equipment for conventional PCR-RFLP [18].

### Sample Collection

Rash illness outbreak is a recurrent problem in DR Congo and has regularly been reported in West Kasai. Accordingly, the way to improve the management of rash illness outbreaks by using rapid on-site DNA-based tests was discussed in a preparatory meeting held end 2007 in Kinshasa with Congolese medical partners and WHO representatives. The decision was to use blotting papers Whatman 903 (GE Healthcare, UK) and sterile 15 ml polystyrene conical screw cap centrifuge tubes (Greiner, Belgium) and to store them in the Kuya Kumpala military camp in Kananga. Following first reports of rash illness outbreak (end January 2008) this material was dispatched to first local healthcare personnel for sample collection on-site. Biological specimens consisted in exudates from vesicular and/or pustular lesions on blotting papers, as well as crusted scabs. After informed consent, human specimens were collected from patients with skin rash disease. For collecting specimens, first responders were instructed to gently apply blotting papers onto exuding vesicular or pustular lesions at 6 different parts on the body and allow the exudates to soak in the filter for at least 60 seconds and then allow the filters to dry. In patients at the stage of recovery, scabs were scraped from healing lesions and stored in dry 15 ml conic tubes (VWR, Belgium). To avoid cross-contamination, each single blotting paper was put in a plastic bag. All plastic bags containing blotting papers were packed in sealed boxes with a double barrier, according to IATA regulations and directly brought to the deployed laboratory by the local WHO representative, as agreed upon in 2007. For each patient, data such as sex, age, date of sampling and setting were carefully recorded. Boxes were opened in a single-use glove box isolator by laboratory operators wearing protective personal equipment including mask and gloves [18].

### DNA Extraction and Molecular Assays

**Figure 1:**
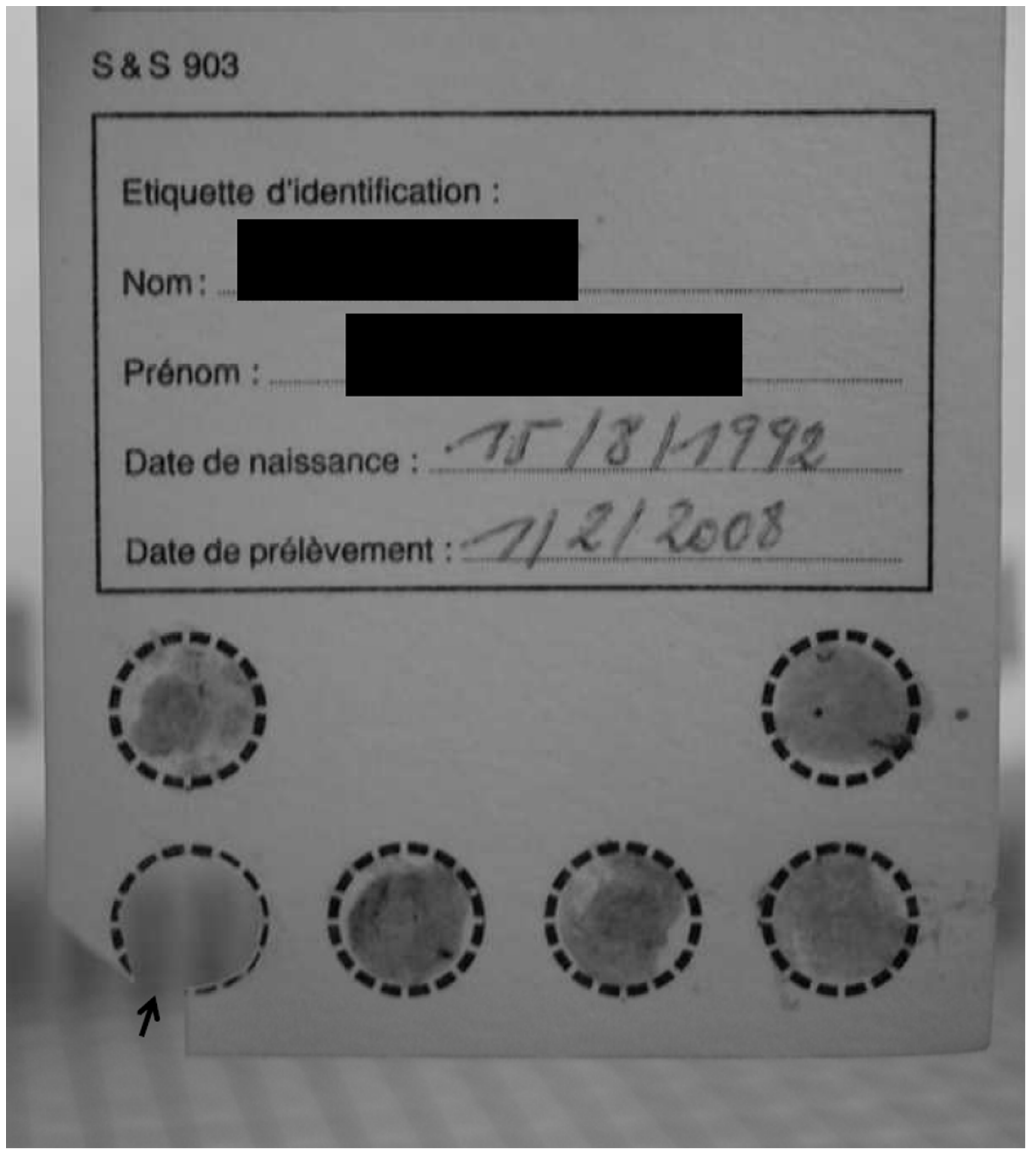
Blotting papers showing six 6 spots with dried liquid exudate from pustules collected in one patient. One spot (arrow) was removed and processed for DNA-based identification of causative agents of the rash illness.

A single spot of blotting paper was cut and DNA extracted (Figure 1) using the RTP DNA/RNA Virus Mini Kit (Invitek, Berlin, Germany) according to the manufacturer’s protocol. For each patient, three different spots were processed separately. DNA was then amplified as described hereafter.

### Development of a qPCR Assay for Generic Detection of Orthopoxviruses and Specific Detection of Smallpox

**Figure 2:**
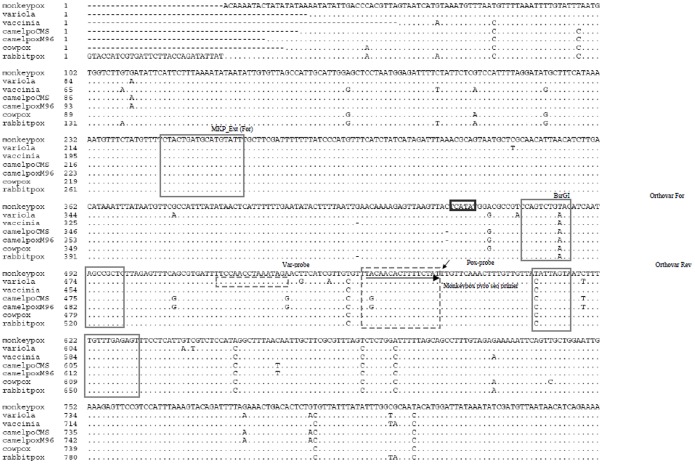
Multiple alignment of 14 kD DNA targets and design of primers and probes for the qPCR assay. Primers for qPCR and for the conventional PCR are shown in plain boxes whereas the pan-orthopoxvirus and specific variola virus probes are highlighted in a dashed boxes. The monkepoxvirus specific SNP (C→T) is arrowed. The pyrosequencing probe is indicated by a plain arrow.

The qPCR assay developed for the detection of clinically relevant orthopoxviruses was a modification of the assay described by Scaramozzino [19]. The modification was aimed at developing a more straightforward assay with respect to data analysis. In brief, after multiple alignments of available orthopoxviruses 14-kilodaltons protein genes (Figure 2), a couple of consensus primers (orthopox-FOR: 5′-ccagagatatcatagccgctctt-3′; and orthopox-REV: 5′-gaaactctcaaacaacgrctaact -3′) were designed to generically amplify a 157-bp fragment of the clinically relevant orthopoxviruses 14-kilodaltons (14-kDa) gene (accession number AF380138.1). The reverse primer was 5′-biotinylated in order to allow subsequent amplicon capture on streptavidin-coated beads for subsequent pyrosequencing assay. A pan-orthopoxvirus 5′ nuclease probe (Pox-probe) (5′-tttgttcaaactttgttgtta-3′) exactly matching a consensus sequence within the 157-bp 14 kDa amplicon was designed for detecting the whole clinically relevant orthopoxvirus genus whereas a second 5′-nuclease probe (5′-taaatagaacgtcatcatt-3′) encompassing a smallpox-specific SNP (Var-probe) was designed for specific detection of smallpox virus. Specificity of primers and probes was assessed *in silico* against all publicly available nucleotide sequence databases by using BLASTN [20]. The Pox- and the Var-probes were respectively labeled with VIC and FAM reporters at their 5′-end. Both probes were labelled at their 3′-end with a MGB group and a non-fluorescent quencher (NFQ). Primers were purchased from Eurogentec (Ougrée, Belgium), whereas probes were purchased from Applied Biosystems (Carlsbad, CA, USA).

Each qPCR assay was carried out in 25 µL of a reaction mixture containing 2.5 µL of extracted DNA as template, 300 nM of each primer and 100 nM of each probe and 12.5 µL of Universal PCR Master Mix (Applied Biosystems, Foster City, USA). The reaction was initiated at 50°C for 2 min, and 95°C for 10 min followed by 45 cycles of denaturation at 95°C for 15 s and annealing/extension at 60°C for 1 min and data were recorded as Cycle threshold (Ct) on a StepOnePlus Real-time PCR system (Applied Biosystems, Foster City, USA), using the analytical software from the same manufacturer.

The orthopoxvirus duplex qPCR assay was first assayed on positive controls, which consisted in recombinant pGEM-T Easy plasmids carrying an insert the 14-kDa fusion protein gene of variola major virus (pVARV-A30L), camelpoxvirus (pCAMV), cowpoxvirus (pCPXV-V162), and MPXV (pMPXV-A29L), as described by Scaramozzino [19]. The specificity of the assay was assessed on DNA isolated from a series of unrelated microorganisms as described previously [21] and on various badges of human genomic DNA samples.

Standard curves were generated from serial dilution of a solution pVARV-A29L (ranging 10^9^ to 1 copy) diluted in a solution containing 40 ng of human DNA. The plasmid copy number was calculated by standard methods using the Avogadro constant and the molecular weight of the recombinant plasmid. These ten-fold serial dilutions were submitted to qPCR and obtained Ct values were plotted against the logarithm of copy numbers to generate a calibration curve. In order to generate calibrations curves, three different qPCR assays were performed. Each DNA solution was assayed in triplicate per qPCR assay. The calculated Ct value corresponding to the intercept (which represents 1 copy of OPV) was arbitrarily set as the threshold for negatives samples. Accordingly, the lowest Ct value of the intercept (95% confidence interval [CI]) was considered as a threshold of negativity for each sample.

### OPV/MPXV Sample Analysis by qPCR

- Pre-validation step: prior to assaying true human specimens in DR Congo, the assay was first tested in the Center for Applied Molecular Technologies (CTMA), Brussels, on soil matrix samples spiked with γ-inactivated class A microorganisms and with vaccinia virus. These samples were obtained in the frame of round-robin biological agents identification exercises which are regularly carried out by NATO countries members [22].
- On-site DNA analysis in Kananga: extracted DNA samples were immediately assayed on-site for OPV detection using the portable StepOnePlus™ qPCR system.

### Conventional MPXV-specific PCR Restriction Fragment Length Polymorphism (RFLP)

A conventional PCR-RFLP was designed as a back-up method for in-field detection of MPXV. In brief, an additional 14 kDa forward primer (MPXV-Ext: 5′-tgatgcatgtatttgcttcgat-3′), located 227- bp upstream of the OPV-var forward primer in the 14-kDa protein gene was used with the OPV-REV primer amplifying a consensus 384-pb fragment. This 384-bp amplicon harboured a MPXV-specific single nucleotide polymorphism A→T in 14 kDa gene at position 139591 in the MPXV genome creating a MPXV-specific *BsrGI* restriction enzyme site. The PCR mixture (50 µL) consisted of 1.5 mM MgCl_2_, 10 mM Tris-HCl pH 8.3, 50 mM KCl, 250 µM of each deoxynucleoside triphosphate (Roche Diagnostics GmbH, Mannheim, Germany), 0.75 U of Taq DNA Polymerase (AmpliTaq Gold for (Roche Molecular Systems Inc., Branchburg, New Jersey, USA), 0.2 µM of each primer and 5 µl of gDNA template. After an initial denaturation step (7 min at 94°C), 40 cycles of amplification were performed in a DNA thermal cycler (Applied Biosystems, Model 2400) as follows: denaturation at 94°C for 1 min, annealing at 60°C for 30 seconds, and primer extension at 72°C for 30 seconds. A final extension was performed at 72°C for 10 min to allow final extension of the incompletely synthesized DNA. The amplified DNA fragments were run in a 2% (w/v) agarose gel, stained with ethidium bromide (0.6 µg/mL), and visualized on a UV transilluminator.

### Restriction Enzyme Analysis

*BsrGI* enzymatic restriction of the MPXV 384-bp amplicon yielded 210-bp and 174-bp fragments, whereas no restriction site was found in OPV amplicons other than MPXV.

After on-site PCR amplification, the concentration of PCR product was estimated using ethidium bromide staining of agarose gels. *BSrGI* restriction enzyme (NEB, Massachussets, USA) was performed by adding 20 units of the enzyme to approximately 1 µg of DNA/PCR product in NEB buffer 2 that had been adjusted to contain 50 mM NaCl, 10 mM Tris-HCl pH 7.9, 10 mM MgCl2, and 1 mM dithiothreitol, and supplemented with bovine serum albumin at the concentration of 1 mg/mL. The mixture was incubated at 37°C during 1 hour. Digested amplicons were resolved on a 2% agarose gel and visualized using ethidium bromide under UV illumination as reported above. A molecular weight ladder was included in each run (100-bp ladder, Invitrogen).

### Molecular MPXV Identification Criteria

A sample was considered as MPXV positive if the following criteria were met: (a) generation of a fluorescence signal with Ct value lower than the lowest Ct of the intercept [CI: 95%] and absence of smallpox-specific signal; (b) generation of a specific restriction banding pattern.

It is of note that the latter condition was further reassessed in a post-deployment phase where pyrosequencing was carried out on all on-site produced biotinylated amplicons. The presence of the MPXV-specific SNP in the 14-kDa gene was assessed and a positive result was defined as a high-quality 100% matching read (see MPXV pyrosequencing hereafter).

### Pyrosequencing of the qPCR Amplicon (Post-deployment Phase)

The pyrosequencing assay was carried out at arrival in CTMA as direct post-deployment analysis and confirmation step in order to reassess all biotinylated amplicons generated on–site. The aim was to test the samples for potential false-negative and -positive results. Regarding the latter issue, attention was paid to each sample defined on-site as MPXV-positive according to the OPV fluorescence signal by qPCR and the MPXV-specific banding pattern by PCR-RFLP. The assay aimed at identifying the MPXV-specific C→T substitution in the 14 kDa gene at position 139716 of the MPXV genome (accession number AF380138.1). Accordingly, the 157-bp 14 kDa biotinylated PCR amplicon were first immobilized on streptavidin sepharose beads, annealed with the MPXV sequencing primer and subjected to pyrosequencing using a pyrosequencer PSQ 96MA (QIAGEN Benelux B.V., Venlo, The Netherlands) according to the manufacturer’s instructions. Following enzyme and substrate incorporation, nucleotides were dispensed in the following order (G, T, C, G, A, T, T, A, G, T, A, A, T) that was derived from the orthopoxvirus sequence alignment (see Figure 2).

### Ethics Statement

Human specimens were taken with the informed consent of all patients. For children, the informed consent was obtained from their parent or guardian. Given the low level of literacy, only verbal consent was sought and obtained. This verbal consent was recorded, prior to sampling, by local first-line responders. Healthcare workers and physicians signed the following statement: “We have explained the study to the patient in the healthcare districts under investigation and are satisfied that he/she understands and consents to sampling”. Ethical approval to conduct the study was obtained from LNSP of the Ministry of Health of DR Congo and the ACOS & Training (Belgian Ministry of Defense). The use of oral consent was approved by the Institutional Review Board of Université catholique de Louvain/Saint-Luc Hospital.

## Results

### Set Up of the Assay and Limit of Detection (LOD)

**Figure 3:**
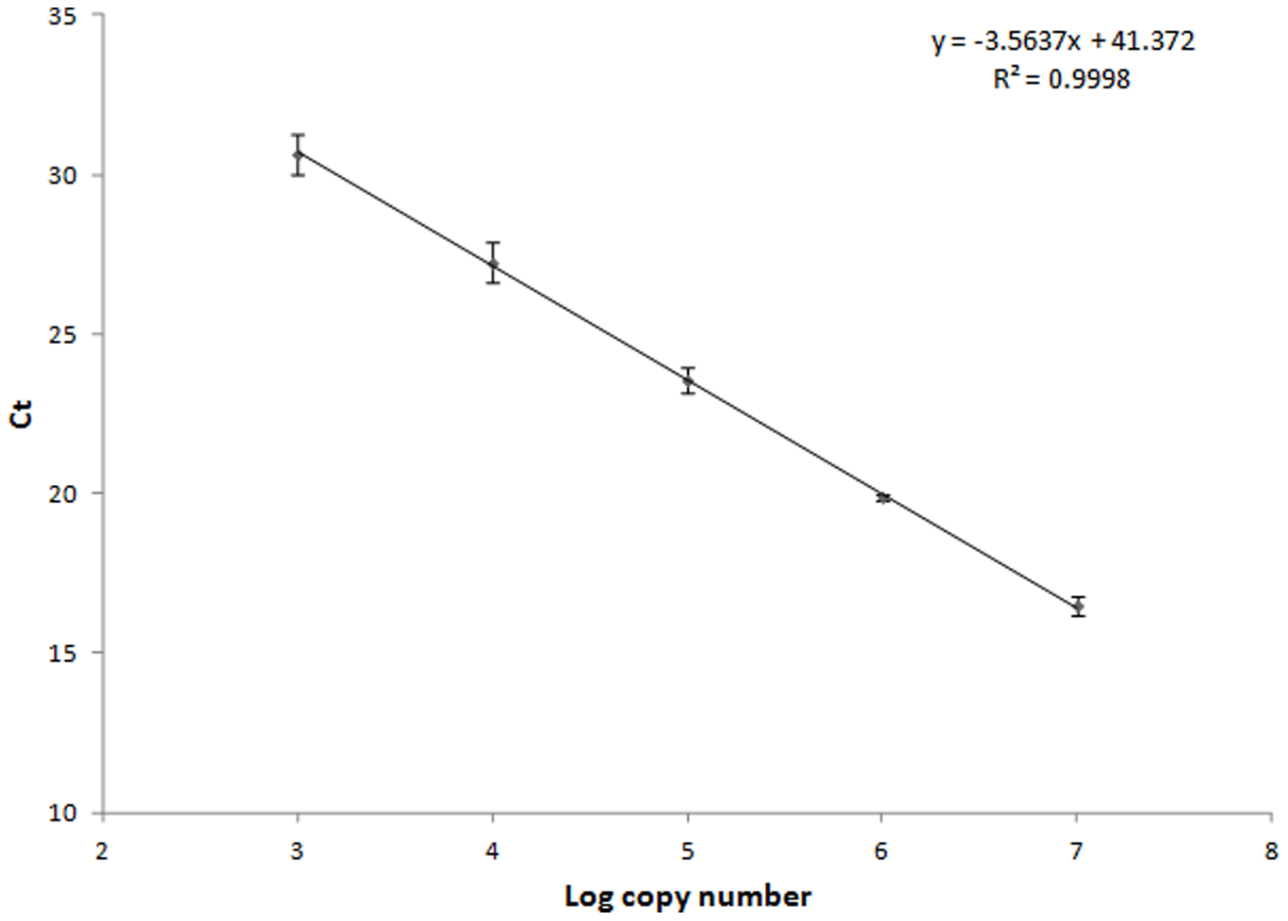
MPXV qPCR amplification standard curve.

The qPCR duplex OPV assay was specific in detecting all clinically relevant OPV, as demonstrated by amplification of positive OPV plasmids and soil samples spiked with inactivated vaccinia virus, while remaining negative in DNA isolated from other microorganisms, as well as with human DNA. Standard curves for the qPCR generated with the MPXV positive control plasmid (pMPXV-A29L) displayed a dynamic range of 5 log DNA dilutions (Figure 3). The calculated Ct value at the intercept (which corresponds to 1 genome copy) was of 41.2, [confidence interval at 95%: 39.8–42.4].

Accordingly, Ct values lower than the 39.8 were considered as potentially positive and subjected to further confirmatory steps as stated in Materials and Methods.

### OPV, MPXV and VZV On-site Identification in Human Samples

In total, 29 clinical specimens from 25 patients presenting with symptoms presumably attributed to MPX were assayed. For each of the four following patients (numbered 03, 05, 08 and 10), two types of specimens were collected including scabs from healing lesions and exudates on blotting papers. Among these patients, 13 originated from 8 different villages within the Bena Tshiadi healthcare district, whereas 9 patients originated from the same village (Tshikongo) in the Yangala healthcare district. Three additional cases with a suspicion of MPX originated from the same village in Ndesha healthcare district (located on the outskirts of Kananga).

**Figure 4:**
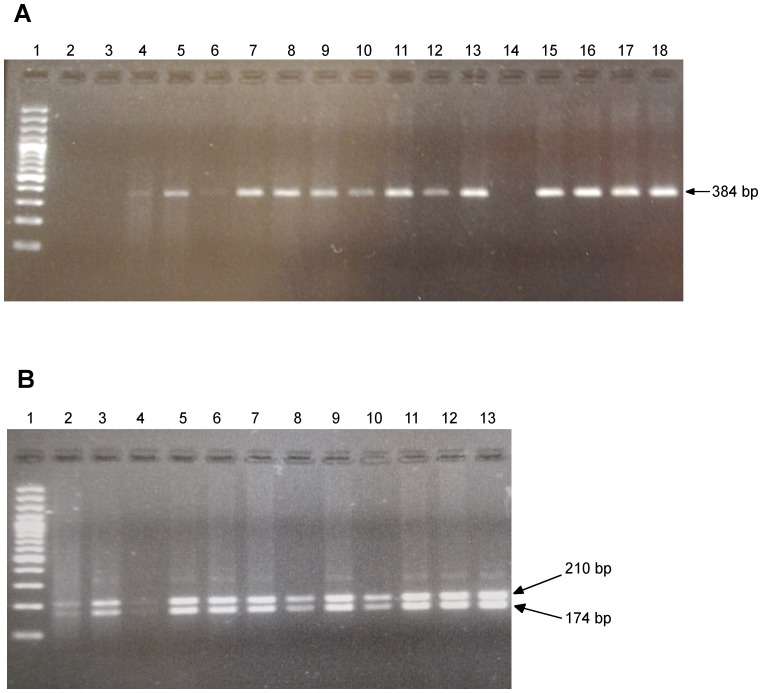
**A)** Gel electrophoresis of the conventional PCR amplification products showing the undigested 384-bp OPV amplicon DNA ladder (lane 1); negative controls (lanes 2–3); human specimens from 13 patients of the Bena Tshiadi healthcare district (lanes 4–13 and 15–18), and from 1 patient of the Yangala healthcare district (lane 14). **B)** *BsrGI* digestion profile of 384-bp MPXV amplicons: banding patterns with 210- and 174-bp fragments.

**Figure 5:**
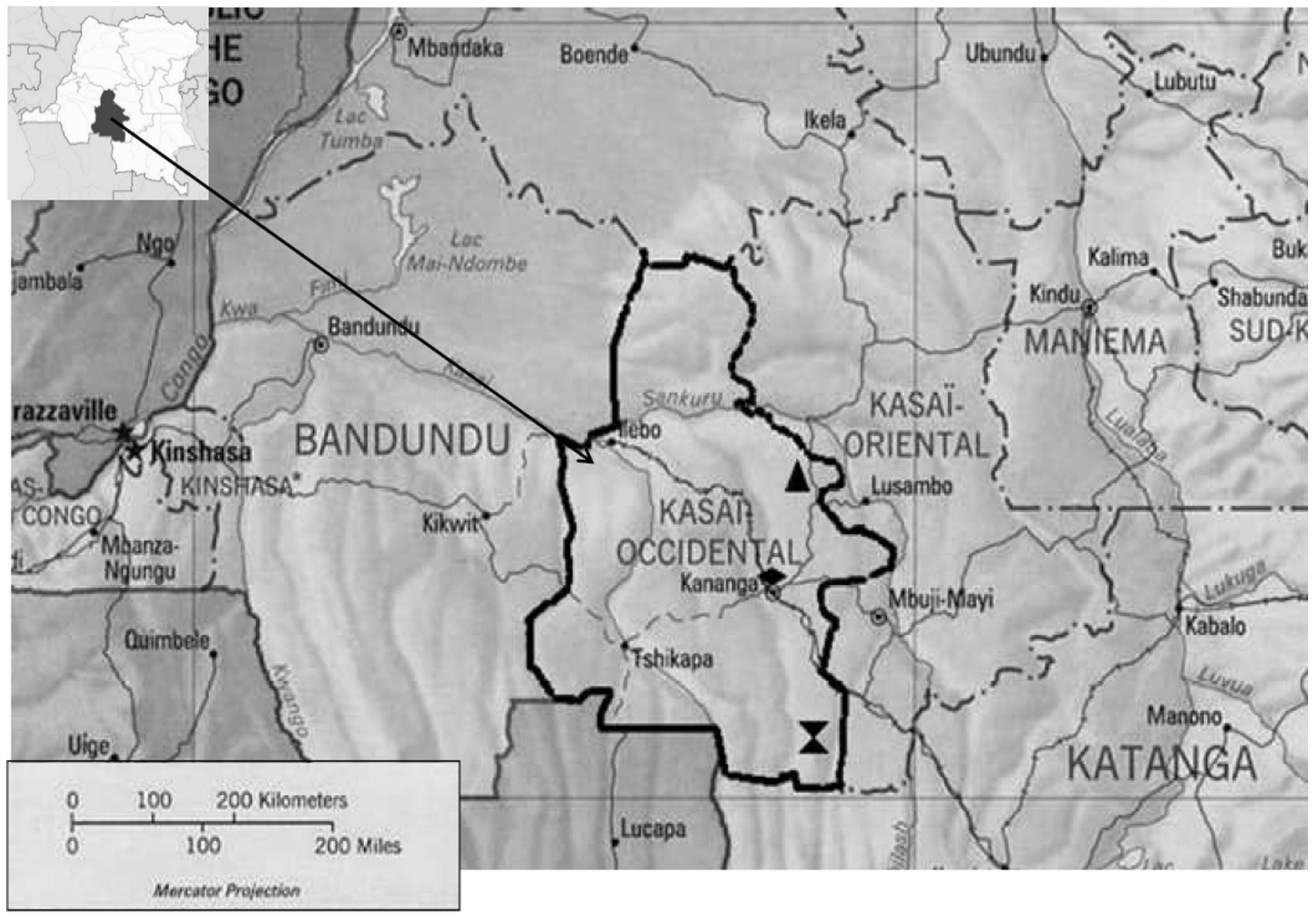
Map of West Kasai Province. Areas affected by rash illness outbreaks are highlighted as follows: ▴: Bena Tshiadi healthcare zone (13 patients in 7 different villages). All cases were confirmed as monkeypox cases. Hourglass Symbol: Yangala healthcare zone (8 patients clustered in Tshikongo village). All were confirmed as varicella cases.♦: Ndesha healthcare zone in the outskirts of Kananga (3 patients clustered in Lubuyi village, several kilometers north of Kananga city). All were confirmed as varicella cases.

No smallpox-positive signal was recorded among all clinical specimens assayed during the deployment and all specimens of patients (n = 13) originating from Bena Tshiadi healthcare district displayed a positive pan-orthopoxvirus signal compatible with MPX. Subsequent on-site identification of MPXV by *BSrGI* RFLP displayed a MPXV-specific banding pattern (Figure 4). The validity of the results was further confirmed by pyrosequencing analysis of the biotinylated amplicons and identification of the presence of the MPXV virus-specific C→T SNP at position 139716 of the 14-kDa protein gene. In 12 remaining pan-OPV-negative specimens, the test for the presence of VZV DNA was positive. Among these 12 VZV-positive patients, 9 resided in Tshikongo village (Yangala healthcare district), whereas 3 originated from Ndesha healthcare district. A map of the Kasai-Occidental showing the areas affected by the different rash illness outbreaks is shown in Figure 5. The turn-around time for the assay (from sample reception) to provisional identification was 5 hours. These provisional results were transmitted to the WHO local representative in Kananga, who conveyed them to the LNSP.

## Discussion

This study assessed the feasibility of establishing a rapid, specific and strict discrimination between rash illnesses outbreaks caused either by monkeypox or varicella viruses while also assessing concomitantly the presence of clinically relevant OPV including specifically smallpox. This was carried out by on-site analysis of blotting papers collected in very remote areas and soaked with exudates from pustular lesions, or by scrub analysis. Whereas official eradication of smallpox could reasonably exclude smallpox as a part of this differential diagnosis, CRBN contingency plans set up all around the world do not rule out a criminal use by terrorists or rogue nations. Consequently, integrating this assay in a dual-use strategy which combines preparedness against biological weapon and natural outbreaks remains highly relevant, irrespective of smallpox eradication. Regarding MPX and varicella, both diseases are known to coexist in Africa and are easily confused, as would also be smallpox in remote endemic African areas with weak primary health care delivery system and poor economic resources. Considering the very negative impact of the latter factor on the level of education, it is not surprising that clinical features of MPX such as a febrile prodrome are often overlooked in these regions. Furthermore, the visual evaluation of skin lesions can be confusing, especially for first health responders. An illustration of this challenge is highlighted by patients 03, 05, 08 and 10 who all displayed PCR-positive signals for MPX while presenting concomitantly pustular lesions and scabs, a feature rather associated with varicella.

The rationale of this study was therefore to consider the West Kasai province as a test case to demonstrate the feasibility of providing rapid on-site DNA-based differential diagnosis between smallpox, monkeypox and varicella, hence to achieve a dual use of identification tests targeting bioterrorism agents as well as agents involved in natural outbreaks. These DNA analyses were carried out in a rapidly transportable and deployable facility through the processing of clinical specimens which were all smoothly and safely collected and transported by first responders, despite a lack in highly specialized training and medical support infrastructure.

Blotting papers proved to a very simple sampling method, easy to implement in remote areas such as those found in West Kasai. This province is indeed composed of several lowly populated villages scattered in an area spanning on more than 2500 Km^2^. In that respect, it illustrates perfectly the many issues related to rapid identification and management of a broad range of infectious disease causing agents among which those involved in outbreaks of skin rash illness or haemorrhagic fever represent only the tip of the iceberg. The lack of a well-organised and efficient transportation network for shipping specimens to the central reference facility in Kinshasa hampers indeed a rapid assessment of outbreaks and medical conditions related to endemic and epidemic diseases. In addition, appropriate supplies necessary for collecting samples in the region, cold-chain for storage and transportation of clinical specimens, as well as personnel trained to perform blood collection or other interventions are all lacking. These particularly challenging issues are some of the major hindrances which need to be dealt with by local healthcare authorities when considering management and mitigation of the medical consequences of endemic and epidemic infectious diseases.

Regarding the type of biological specimens to select, it was anticipated that collecting clinical specimens other than blood samples with the purpose of testing them locally by using rapid DNA-based assays would be the most straightforward and appropriate process. Referring to past history, it is indeed worth reminding that antibodies-based immunological assays for detecting MPX and varicella were previously reported by others in a similar context [12]. However, the local conditions prevailing during the West Kasai outbreak had to be carefully considered, as would also be requested in any difficult work environment. In such challenging circumstances, these methods may be inappropriate as they require blood sampling via skin or finger puncture, hence the contribution of healthcare personnel, appropriate hygiene conditions, adequate storage and transportation. Accordingly, collecting vesicular and/or pustular exudates on blotting paper for subsequent DNA-processing appeared as the most straightforward, reliable and simplest alternative. The ground principle for choosing this method was also two original reports demonstrating the presence of variola virus in exudates [23] and the presence of MPXV in epidermal cells [24], therefore suggesting the presence of the rash causative viruses in pustules. Moreover, exudates soaked onto blotting paper or scabs taken during the healing process of patients with contagious skin rash have also proved to be appropriate for easy and low-cost sampling, for safe transportation even in rough conditions, and for long term (years) conservation at room temperature. The latter point was demonstrated by successful electron microscopy analysis and qPCR of current samples four years after collection (personal data). Accordingly, sampling pustules or vesicles with purulent exudates was carried out by gently applying blotting papers onto these lesions, a procedure which requires neither specific skill nor medical supervision.

While a range of molecular methods, among which several qPCR assays, were previously reported to identify orthopox viruses [13], [19], [25]–[33], pyrosequencing-based detection of SNP for identification of MPXV is, to the best of our knowledge, reported here for the first time. Yet, pyrosequencing is expected to provide more reliable results than would qPCR using a probe or PCR-RFLP. Pyrosequencing results consist indeed in high quality sequences of short stretches of DNA. A dual objective underpinned therefore the use of pyrosequencing as a complementary method for the current on-site diagnostic approach: whereas the fieldable analytical instrumentation (i.e., performing qPCR and PCR-RFLP) allowed to achieve rapid and provisional on-site identification, unambiguous identification of the causative agent would be required by reference laboratories. As unambiguous identification of agents causing outbreaks or CBRN incidents is classically performed in reach back laboratories using appropriate culture and DNA sequencing, we therefore aimed to test pyrosequencing as confirmation method [34]–[36]. Although not assessed yet as first line diagnostic method in field conditions, the pyrosequencer has several features making it suitable as field analyzer (i.e., small volume, low weight, easy use, accuracy and automated reading). The advantage of this method is its ability to provide good quality sequence of selected DNA fragments hence paving the way for on-site unambiguous identification with a quality equal to this offered by a reference laboratory. As demonstrated in this work, it also offers the very interesting possibility to sequence directly all amplicons generated by qPCR, provided that biotinylation of these amplicons has been carried out. Meanwhile, new features and improvements have been introduced to optimize the use of this equipment for in-field applications, especially through novel multiplexing analytical capacity [37]. The interest of having a local sequencing capacity at disposal during outbreaks of infectious diseases could indeed be paramount: besides the threat of smallpox as biowarfare agent, the emergence of novel OPV diseases are a real concern due to waning immunity in populations previously vaccinated against variola and to the poor state of healthcare delivery system [38].

Regarding the PCR-RFLP assay, this is not an optimal method for in-field testing. Accordingly, it should not be recommended for this use but rather be replaced by new emerging technologies circumventing or decreasing the risk of carry-over contamination. Nevertheless, this assay was considered here as a “back up method” in case of qPCR breakdown. It was easily carried out, did not request any sophisticated pieces of equipment and, as presented hereunder, provided results which were all confirmed by pyrosequencing after our return to Belgium.

Regarding the biological results obtained on-site by qPCR and PCR-RFLP, and confirmed in CTMA by pyrosequencing, a total of 29 samples collected from 25 patients were analyzed for identifying agents causing the rash illness. Although the selected patients were all presumably clinically diagnosed with MPX, it turned out that only 13 of them tested positive for MPXV whereas 12 tested positive for VZV. This observation is consistent with previous reports that VZV infection is commonly mistaken as being MPX in MPX-endemic regions [12], [14]. Accordingly, DNA-based MPXV and VZV differential diagnosis carried out on-site or as close as possible of the affected sites appears as a highly relevant strategy. While age and gender were comparable among patients (Table 1), it is interesting to note that their geographical location differed strikingly with VZV clustering to a single village in the Yangala as well as in the Ndesha healthcare districts. In contrast, MPX scattered throughout the outbreak area. This observation is consistent with a higher rate of human-to-human VZV transmission.

**Table 1:** DNA-based identification of causative agents of rash illness outbreaks (2008 and 2009) in West Kasai province.

Patient (N°)	Sample type	Age (years)	Residence (village)	Sampling date	OPV fluorescence signal(Mean Ct +/− SD)	VZV fluorescence signal(Mean Ct +/− SD)	MPXV SNP (14-kDagene C139716T)	BsrGIRestriction	Diagnosis
1	Blotting paper	6	Bena Nkana	1/02/2008	16,88±0,46	Undetermined (Ct value >45)	Present	Yes	MPXV
2	Blotting paper	7	Diloba	30/01/2008	17,33±0,45	Undetermined (Ct value >45)	Present	Yes	MPXV
3	Blotting paper	33	Mbiyi Diloba	2/02/2008	31,85±0,10	Undetermined (Ct value >45)	Present	Yes	MPXV
3	Crust				14,45±0,15	Undetermined (Ct value >45)	Present	Yes	MPXV
4	Blotting paper	10	Bena Mbebe	2/02/2008	15,42±0,06	Undetermined (Ct value >45)	Present	Yes	MPXV
5	Blotting paper	8	Bakua Tambua	2/02/2008	33,45±0,32	Undetermined (Ct value >45)	Present	Yes	MPXV
5	Crust				15,99±0,17	Undetermined (Ct value >45)	Present	Yes	MPXV
6	Blotting paper	18	Bena Tshipanga	2/02/2008	17,28±0,66	Undetermined (Ct value >45)	Present	Yes	MPXV
7	Blotting paper	5	Bakua Tambua	2/02/2008	29,97±0,06	Undetermined (Ct value >45)	Present	Yes	MPXV
7	Crust				23,69±2,91	Undetermined (Ct value >45)	Present	Yes	MPXV
8	Blotting paper	31	Bena Nkana	2/02/2008	16,17±0,43	Undetermined (Ct value >45)	Present	Yes	MPXV
8	Crust				19,39±0,74	Undetermined (Ct value >45)	Present	Yes	MPXV
9	Blotting paper	30	Bisabi	2/02/2008	30,63±0,08	Undetermined (Ct value >45)	Present	Yes	MPXV
10	Blotting paper	16	Bisabi	2/02/2008	16,81±0,25	Undetermined (Ct value >45)	Present	Yes	MPXV
10	Crust				23,06±0,99	Undetermined (Ct value >45)	Present	Yes	MPXV
11	Blotting paper	6	Bena Mbebe	1/02/2008	18,44±0,20	Undetermined (Ct value >45)	Present	Yes	MPXV
12	Blotting paper	34	Diloba	1/02/2008	18,9±0,07	Undetermined (Ct value >45)	Present	Yes	MPXV
13	Blotting paper	5	Bena Tshipanga	29/01/2008	16,1±0,21	Undetermined (Ct value >45)	Present	Yes	MPXV
14	Blotting paper		Tshikongo	2/08/2008	Undetermined (Ct value >45)	31.48±0.23	ND	ND	VZV
15	Blotting paper		Tshikongo	28/07/2008	Undetermined (Ct value >45)	30.79±0.70	ND	ND	VZV
16	Blotting paper		Tshikongo	4/08/2008	Undetermined (Ct value >45)	30.26±0.59	ND	ND	VZV
17	Blotting paper		Tshikongo	25/07/2008	Undetermined (Ct value >45)	29.14±0.21	ND	ND	VZV
18	Blotting paper		Tshikongo	1/08/2008	Undetermined (Ct value >45)	29.08±0.53	ND	ND	VZV
19	Blotting paper		Tshikongo	30/07/2008	Undetermined (Ct value >45)	34.78±0.88	ND	ND	VZV
20	Blotting paper		Tshikongo	28/07/2008	Undetermined (Ct value >45)	32.07±0.73	ND	ND	VZV
21	Blotting paper		Tshikongo	4/08/2008	Undetermined (Ct value >45)	32.19±0.47	ND	ND	VZV
22	Blotting paper		Tshikongo	4/08/2008	Undetermined (Ct value >45)	31.17±0.37	ND	ND	VZV
23	Blotting paper		Lubuyi	3/04/2009	Undetermined (Ct value >45)	18.27±.0.02	ND	ND	VZV
24	Blotting paper		Lubuyi	4/03/2009	Undetermined (Ct value >45)	24.64±0.08	ND	ND	VZV
25	Blotting paper		Lubuyi	22/02/2009	Undetermined (Ct value >45)	26.12±0.43	ND	ND	VZV

Legend: Patients originated from the following healthcare districts: Bena Tshiadi, 1–13; Yangala, 14–22; Ndesha, 23–25.

To the best of our knowledge, it is the first time that this proof-of-concept was carried out in the healthcare districts of West Kasai with the aim to provide a clear and local diagnosis of a rash illness outbreak. In the current study, no gold standard method was locally available. The concordance between qPCR, RFLP and pyrosequencing results was however perfect. As briefly mentioned above, it is interesting to add that electron transmission microscopy carried out a year later on filter papers also confirmed the presence of viral-like particles morphologically compatible with OPV in MPXV-positive patients. While the latter analysis was not intended to provide a viral-specific diagnosis, the agreement between these different methods ascertained the quality of results generated in the field.

In conclusion, this proof-of-concept study underscores the potential for implementing a straightforward sampling method combined with DNA-based methods for identification of some causative agents of rash illness outbreaks in remote areas.
